# Rotating Sonotrode Design for Ultrasonic-Assisted Arc Welding of Metal Materials

**DOI:** 10.3390/ma17071599

**Published:** 2024-03-31

**Authors:** Xinyu Mao, Zhidong Yang, Qihao Chen, Mingzhu Hu, Tian Gan

**Affiliations:** 1 Provincial Key Laboratory of Advanced Welding Technology, Jiangsu University of Science and Technology, Zhenjiang 212003, China; mxy15950314480@163.com (X.M.); yangzhidong@just.edu.cn (Z.Y.); m_z_hu@163.com (M.H.); 2 Nanjing KathMatic Technology Co., Ltd., Nanjing 211100, China; reesegan@mumuxili.com

**Keywords:** rotating sonotrode with a groove, ultrasonic, welding, amplification coefficient

## Abstract

In the process of the ultrasonic-assisted arc welding of metal materials, traditional ultrasonic application methods, such as the low-frequency impact of ultrasonic horns on a base material, can easily cause the non-fusion defect. In order to solve this problem, a rotating sonotrode with a groove and double thin ends was designed in this study. The ultrasonic vibration is transmitted into the weld pool by the rolling of the sonotrode on both sides of the weld. The resonant frequency was set at 50 kHz. Firstly, based on the Mindlin theory, a rotating sonotrode without a groove was designed by solving the frequency equation and by conducting a finite element simulation. Secondly, the effects of the groove, perforation, and transition mode on the resonant frequency, stress distribution, and amplification factor were investigated by finite element simulation. Finally, the optimum rotating sonotrode with a groove was obtained. The results show that the size of a rotating sonotrode that has a small frequency error can be obtained by using the discrete interval solver method combined with finite element simulation. The groove can significantly reduce the resonant frequency. The stress concentration can be effectively reduced by using the elliptical transition mode. The resonant frequency and amplification factor of a rotating sonotrode with a groove could be effectively adjusted by a method of double-position joint perforation. The final resonant frequency was 49.721 kHz and the amplification factor was 3.02. This study provides an effective design method for a sonotrode with double thin ends and a groove structure.

## 1. Introduction

There is an important application value for ultrasonic technology in the arc welding of metal materials. Ultrasonic energy can be used to refine the weld microstructure [1], reduce weld porosity defects [2], and increase weld width and depth [3], through which the mechanical properties of the welded joint can be enhanced [4]. Ultrasonic energy can also improve the corrosion resistance of welded joints [5]. Yang proposed a method of ultrasonic impact during arc welding that involves applying periodic ultrasonic energy to the weld pool, which could reduce the input of ultrasonic energy and improve the weld structure. Results have demonstrated that periodic ultrasonic energy can break grains and promote heterogeneous nucleation [6]. Low-frequency mechanical impact is introduced in the process of ultrasonic impacts during arc welding, which can lead to a detrimental effect on welding quality, such as incomplete fusion defects in the weld, decreased weld width and penetration, coarse grains, and decreased mechanical properties [7]. In order to mitigate the adverse effects of mechanical impacts with low frequencies, the introduction method of periodic ultrasonic vibration must be changed. Therefore, it is necessary to design a sonotrode that can roll on both sides of the weld. In this study, periodic ultrasonic power supply is applied, and the periodic ultrasonic energy is transferred from the sonotrode into the weld pool. 

There are usually two methods in the design of sonotrodes: a solution for the frequency domain equation and one for numerical simulation. Yazdian and Hamed designed a booster and composite sonotrode by an analytical method, and they also performed finite element analysis [8,9]. Hornišová demonstrated that the transfer matrix method is more precise than the impedance matrix method and the equivalent circuit method [10]. Yahya designed an acoustic sonotrode for assisted-rotational magnetorheological abrasive flow, which is finished through finite element simulation. The results showed that ultrasonic vibration can reduce the surface roughness of the component [11]. The amplification factor of acoustic sonotrodes is related to the ratio of the cross-sectional areas at both ends of an acoustic sonotrode. The larger the cross-sectional ratio is, the higher the amplification factor. The stepped acoustic sonotrode has a large amplification factor, but stress concentration readily occurs at the variable section of the acoustic sonotrode, and the safety factor of the acoustic sonotrode is low [12,13,14,15]. In order to address the issue of stress concentration at the variable cross-section of the stepped horn, it is usually necessary to design a transition section at the variable cross-section. Zeng clarified the optimal single-arc transition curve for sonotrodes with various cross-section ratios through finite element analysis [16]. Based on single-arc transition, González-Mendoza proposed that the design of a pressure relief groove can decrease the stress concentration [17]. Tu found that the effects of double-curvature arc transition, three-curvature arc transition, elliptical transition, and streamline transition on reducing stress concentration are superior to single-arc transition [18]. Hannemann studied the crack propagation factors on the elliptical transition segment of the stepped shoulder and found that the radius of the transition segment was one of the main influencing factors of crack propagation [19]. Mehran designed a wide-blade ultrasonic horn based on finite element simulation, and the ultrasonic vibrations were amenable to being evenly distributed on the horn [20]. Satpathy designed a stepped sonotrode to weld thin metal plates by using ANSYS R15.0 and COMSOL 5.2a software, and the results showed that the displacement node was the most vulnerable part [21]. Hameed designed a sonotrode with a characteristic frequency that is greater than that of the transducer, and they found that that sonotrode had a larger amplification factor and a smaller stress distribution than the traditional sonotrode [22].

A rotating sonotrode consists of a thin end and a roller end. The design of the roller end mainly relies on the Kirchhoff theory and the Mindlin theory. The design of the thin end mainly relies on the wave equation theory of longitudinal vibration. A rotating sonotrode can be used for ultrasonic textiles [23,24], ultrasonic plastic welding [25], ultrasonic atomization [26], and the ultrasonic cold working of metals. Kustron designed two different ultrasonic atomizing devices with different characteristic frequencies based on finite element simulation for the metal powder process, and the amplitude distribution of the two ultrasonic atomizing devices is also similar [27]. The ultrasonic metal cold working process mainly includes ultrasonic rolling, ultrasonic cutting, ultrasonic gear honing, and ultrasonic grinding. The roller end can be classified into three categories based on their thickness-to-diameter ratio, namely the thin plate, medium plate, and thick plate. The Kirchhoff theory was applied to the design of the roller end thin plate, and the Mindlin theory was applied to the design of the roller end thin plate and medium plate. Zhang employed the Kirchhoff theory to design a rotating sonotrode for the ultrasonic rolling process. The results showed that, compared to the segmented design, it is easy to realize the resonance of an ultrasonic system when the rotating sonotrode is designed as a whole and when the roller end can amplify the amplitude [28]. The machining of a honeycomb composite material is one of the application directions of ultrasonic cutting. The cutting blade is the roller end of a rotating sonotrode, and the characteristic frequency of the sonotrode increases with the increase of blade thickness [29,30,31]. For the ultrasonic gear honing process, the roller end of the sonotrode is usually a gear. The gear is matched with the gear to be machined, and the roughness of the tooth root can be reduced through ultrasonic vibration [32]. The size of the roller end is usually not designed because it is determined by the parameters of the gear being machined. The design of a rotating sonotrode is predominantly intended for the thin end part. In the design, the gear is simplified as a circular plate with a diameter that is identical to that of its dividing circle. Additionally, various thin-end shapes were designed and their sizes were solved. Zhang and Zhou applied the Mindlin theory to design a rotating sonotrode with a conical transition composite thin end by an analytical method [33] and a transfer matrix method [34], respectively. The results demonstrated that the frequency of the rotating sonotrode decreases when there is an increase in the radius of the roller end and increases with an increase in thickness of the roller end [34]. Based on the theory of three-dimensional elasticity and the Chebyshev–Ritz method, the characteristic frequency of a rotating sonotrode composed of a cone and a gear with free vibration was obtained by Li [35]. During the ultrasonic grinding process, the design of the rotating sonotrode was relatively complex, and the thickness and material of the grinding layer on the outer side of the roller end had to be considered. Based on the Mindlin theory, Wu created a rotating sonotrode composed of a three-annular plate-shaped roller end and a conical thin end. The roller end material included brass and gray cast iron, while the thin-end material was 45 steel [36]. On this basis, Fu developed numerical solution software with a MATLAB/GUI interface for a rotating sonotrode, as well as designed a rotating sonotrode with an aluminum-based phenolic resin binder as the roller end material [37].

**Figure 1:**
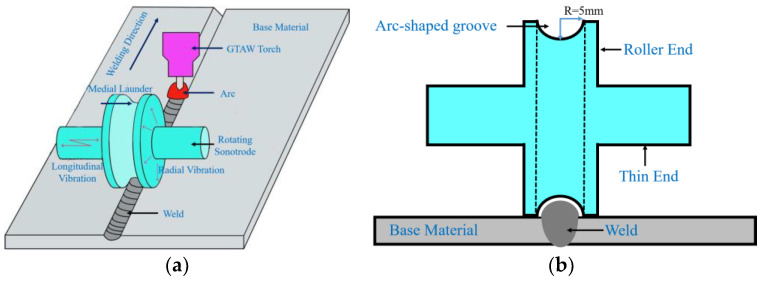
Diagram of the designed rotating sonotrode: (**a**) A three-dimensional diagram and (**b**) a two-dimensional diagram.

This study aims to design a rotating sonotrode that can roll on both sides of the weld during the arc welding process of metal materials. The three-dimensional diagram is shown in Figure 1a. In order to mitigate the impeding effect of a raised weld shape on the rolling of the rotating sonotrode, it was necessary to create an arc groove with a radius of 5 mm on the outer surface of the roller end, as shown in Figure 1b. Both thin ends were subjected to the same pressure so that the roller end could be in close contact with the base material. The frequency of the existing sonotrode was mainly between 20 kHz and 35 kHz. In this study, in order to reduce the size of the rotating sonotrode, the frequency of the rotating sonotrode was designed to be 50 kHz.

**Figure 2:**
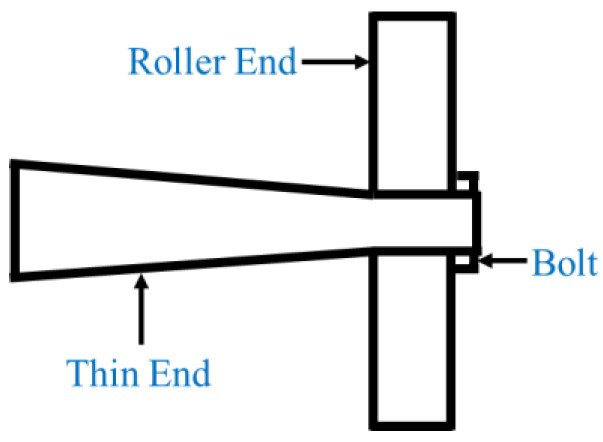
Diagram of the single thin-end rotating sonotrode.

In comparison with the existing research on rotating sonotrodes, the specific differences from this article are as follows:

(1) The application fields and assembly method for a rotating sonotrode are different. The rotating sonotrode developed in the current research was predominantly employed in the field of machining. The rotating sonotrode was assembled by the bolt connection method, as shown in Figure 2. The application direction of the rotating sonotrode designed in this study was used to assist the arc welding process of metal materials, which is composed of double thin ends and a roller end. The rotating sonotrode in this study is a whole unit and does not involve mechanical assembly.

(2) The calculation methods for the rotating sonotrode sizes are different. In previous studies, the size of the roller end is determined, and the size of the thin end needs to be solved. In this study, a double thin-end rotating sonotrode was designed. The size of the fine end was fixed and the size of the rolling end, which is more difficult to solve, was solved.

(3) The shapes of the rotating sonotrode are different. In this study, the rotating sonotrode has a curved groove. The method for making a slotted rotating sonotrode that has a target design frequency and large amplification factor on the basis of satisfying the longitudinal–flexural resonance mode is very critical.

Based on the above analysis, in order to design a rotating sonotrode for the ultrasonic vibration-assisted arc welding process of metal materials, the design went through the following steps: Firstly, a mathematical model of the unslotted rotating sonotrode was established based on the Mindlin theory. TC4 titanium alloy was used as the material of the rotating sonotrode. The size of the rotating sonotrode was solved by MATLAB R2022a software, and the size was optimized by finite element simulation. Secondly, the effect of the transition form of the junction between the roller end and the thin end; the perforation on the roller end on the stress distribution; and the characteristic frequency and amplification factor of the sonotrode were investigated by the finite element simulation method. Finally, by optimizing the position and size of the perforation, the designed rotating sonotrode was able to reach the target design frequency and had a large amplification factor. This study provides an effective design method for the design of rotating sonotrodes with double thin-ended structures.

## 2. The Design Route of Double Thin-End Rotating Sonotrodes

A rotating sonotrode with a longitudinal–flexural resonance mode at a characteristic frequency of 50 kHz was designed. It was imperative to apply pressure at the node position of the thin end in order to guarantee the transmission of the ultrasonic vibration. The node positions at the two thin ends had to be symmetrical relative to the roller end. Therefore, the shape of the thin end could not be too complicated. The two thin ends of the rotating sonotrode were designed as equal cross-section rods, and the length of the thin end was fixed at half of the wavelength. Subsequently, the size of the roller end, comprising the outer diameter, inner diameter, and thickness, was resolved. In this study, the number of unknowns increased, and it was more difficult to solve the sonotrode size directly by the frequency equation. It was only possible to obtain the size of the roller end of the rotating sonotrode by solving the frequency equation numerically.

The size of the unslotted rotating sonotrode can be obtained through solving the frequency equation numerically. There could be the smallest error between the target characteristic frequency and the actual characteristic frequency for unslotted rotating sonotrodes with an optimum dimension, which had a longitudinal–flexural resonance mode. The effect of grooving on the ultrasonic vibration and propagation in the rotating sonotrode was analyzed. The influence of the transition mode at the junction between the roller end and the thin end on the stress concentration of the rotating sonotrode was studied, and then the influence of the size and position of the hole on the characteristic frequency and amplification coefficient was studied. Finally, the design of the target rotating sonotrode was completed.

Finite element simulation was employed to investigate the vibration mode, characteristic frequency, and stress distribution of the rotating sonotrode. The solid mechanics model was employed, and the control equation was as follows:

(1) $$\rho \frac{\partial^{2}s}{\partial t^{2}}=\left(\lambda +2\mu \right)grad\left(divs\right)-\mu rot\left(rots\right).$$

In the formula, *μ* is the shear elastic constant; *λ* is the bulk elastic constant; and *s* is the particle vibration displacement.

The rotating sonotrode’s material (TC4 titanium alloy) was defined as a linear elastic material. According to the different research objectives, the surface of the rotating sonotrode was given as different boundary conditions. The free triangle model was used to mesh the rotating sonotrode, and the interior of the rotating sonotrode was a tetrahedral grid with a maximum unit length of 11.3 mm and a minimum unit length of 1.41 mm.

## 3. Design Theory and the Results of the Unslotted Rotating Sonotrodes

### 3.1. Mathematical Model and Boundary Conditions

**Figure 3:**
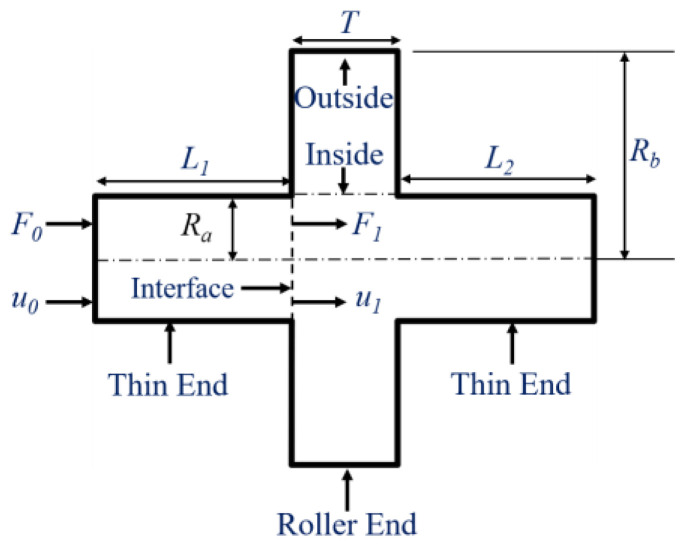
A two-dimensional diagram of the unslotted rotating sonotrode.

The two-dimensional diagram of the unslotted rotating sonotrode is shown in Figure 3. Ra and Rb are the radius of the thin end and the roller end of the rotating sonotrode, respectively. The lengths of L1 and L2 correspond to the respective lengths of the two thin ends of the rotating sonotrode. The thickness of the roller end was defined as T.

The ultrasonic transducer uniformly input the axial force *F*_0_ and axial displacement u0 to the end surface of the thin end, and then the axial force and the axial displacement were transmitted to the interface between the thin end and the roller end, while simultaneously supplying the axial force *F*_1_ and axial displacement *µ*_1_ to the roller end. The transfer matrix on the force and displacement in the thin end is as follows:

(2) $$\left[\begin{array}{c} u_{1}\\ F_{1} \end{array}\right]=\left[\begin{array}{cc} \cos kL_{1} & -\frac{\sin kL_{1}}{kES_{1}}\\ kES_{1}\sin kL_{1} & \cos kL_{1} \end{array}\right]\left[\begin{array}{c} u_{0}\\ F_{0} \end{array}\right]=M_{1}\left[\begin{array}{c} u_{0}\\ F_{0} \end{array}\right].$$

In the formula, *k* is the circular wave number, *k* = 2πf/c, c is the ultrasonic velocity in the rotating sonotrode, and *S*_1_ is the cross-sectional area of the thin end of the rotating sonotrode, where *S*_1_ = π$R_{a}^{2}$.

The roller end of the rotating sonotrode must have a certain thickness to ensure that the slotted roller end can make contact with the base metal on both sides of the weld, which has a high strength. The roller end was set to a medium thickness plate (T: R_b_ = 0.2–0.5). According to the Mindlin theory, the formulas for calculating the deflection, rotation angle, shear force, and bending moment of the roller end of a rotating sonotrode are as follows:

(3) $$w_{r}=\sum_{i=1}^{2}\left[A_{i}J_{0}\left(\delta_{i}r\right)+B_{i}Y_{0}\left(\delta_{i}r\right)\right],$$

(4) $$\beta_{r}=\sum_{i=1}^{2}\left\{\delta_{i}\right.\left(\sigma_{i}-1\right)\left.\left[A_{i}J_{0}^{\prime }\left(\delta_{i}r\right)+B_{i}Y_{0}^{\prime }\left(\delta_{i}r\right)\right]\right\},$$

(5) $$Q_{r}=k_{\tau }Gh\sum_{i=1}^{2}\left[A_{i}\sigma_{i}J_{0}^{\prime }\left(\delta_{i}r\right)+B_{i}Y_{0}^{\prime }\left(\delta_{i}r\right)\right],$$

(6) $$M_{r}=\sum_{i=1}^{2}\left\{A_{i}\left(\sigma_{i}-1\right)\left[J_{0}^{\prime \prime }\left(\delta_{i}r\right)+\frac{\nu }{r}J_{0}^{\prime }\left(\delta_{i}r\right)\right]+B_{i}\left(\sigma_{i}-1\right)\left[Y_{0}^{\prime \prime }\left(\delta_{i}r\right)+\frac{\nu }{r}Y_{0}^{\prime }\left(\delta_{i}r\right)\right]\right\}.$$

In the formula, the values *w_r_*, *β_r_*, *Q_r_*, and *M_r_* represent the deflection, rotation angle, shear force, and bending moment of the roller end of a rotating sonotrode, respectively. Ai and Bi are undetermined constants, whereas *J*_0_, *J*′, and *J*″ are the 0th, 1st, and 2nd derivatives of the Bessel functions of the first kind. *Y*_0_, *Y*_0_′, and *Y*_0_″ are the 0th, 1st, and 2nd derivatives of Bessel functions of the second kind. *G* is the shear modulus, *k_τ_* is the shear influence factor, and *k_τ_* = π^2^/12, *δ_i_*, and *σ_i_* are the defined parameters. The expressions for these are as follows:

(7) $$\delta_{1}=\frac{\delta_{0}^{4}}{2}\left\{R+S+{\left[{\left(R-S\right)}^{2}+\frac{4}{\delta_{0}^{4}}\right]}^{\frac{1}{2}}\right\},$$

(8) $$\delta_{2}=\frac{\delta_{0}^{4}}{2}\left\{R+S-{\left[{\left(R-S\right)}^{2}+\frac{4}{\delta_{0}^{4}}\right]}^{\frac{1}{2}}\right\},$$

(9) $$\delta_{0}^{4}=\frac{\rho T}{D}\omega^{2},$$

(10) $$\sigma_{1}=\frac{\delta_{2}^{2}}{R\delta_{0}^{4}-S^{-1}},$$

(11) $$\sigma_{2}=\frac{\delta_{1}^{2}}{R\delta_{0}^{4}-S^{-1}}.$$

In the formula, *ρ* is the density of the rotating sonotrode material, *D* is the bending stiffness, *R* is the moment of inertia, *S* reflects the influence of transverse shear deformation on the bending deformation of the roller end, and *ω* is the circular frequency of the rotating sonotrode. The expressions for these are as follows:

(12) $$D=\frac{ET^{3}}{12(1-\mu^{2})},$$

(13) $$R=\frac{T^{2}}{12},$$

(14) $$S=\frac{k_{\tau }D}{GT},$$

(15) ω=2πf.

In the formulas, *E* and *μ* are the elastic modulus and Poisson’s ratio.

The Expressions (2)–(5) can be transformed into the matrix form. The matrix forms for the inner part of the roller end (*r* = *R_a_*) and the outer part of the roller end (*r = R_b_*) are as follows:

(16) $$\left[\begin{array}{l} w_{r|r=R_{a}}\\ \beta_{r|r=R_{a}}\\ M_{r|r=R_{a}}\\ Q_{r|r=R_{a}} \end{array}\right]=\left[\begin{array}{l} \begin{array}{cccc} D_{a11} & D_{a12} & D_{a13} & D_{a14} \end{array}\\ \begin{array}{cccc} D_{a21} & D_{a22} & D_{a23} & D_{a24} \end{array}\\ \begin{array}{cccc} D_{a31} & D_{a32} & D_{a33} & D_{a34} \end{array}\\ \begin{array}{cccc} D_{a41} & D_{a42} & D_{a43} & D_{a44} \end{array} \end{array}\right]\left[\begin{array}{l} A_{1}\\ B_{1}\\ A_{2}\\ B_{2} \end{array}\right]=D_{a}\left[\begin{array}{l} A_{1}\\ B_{1}\\ A_{2}\\ B_{2} \end{array}\right],$$

(17) $$\left[\begin{array}{l} w_{r|r=R_{b}}\\ \beta_{r|r=R_{b}}\\ M_{r|r=R_{b}}\\ Q_{r|r=R_{b}} \end{array}\right]=\left[\begin{array}{l} \begin{array}{cccc} D_{b11} & D_{b12} & D_{b13} & D_{b14} \end{array}\\ \begin{array}{cccc} D_{b21} & D_{b22} & D_{b23} & D_{b24} \end{array}\\ \begin{array}{cccc} D_{b31} & D_{b32} & D_{b33} & D_{b34} \end{array}\\ \begin{array}{cccc} D_{b41} & D_{b42} & D_{b43} & D_{b44} \end{array} \end{array}\right]\left[\begin{array}{l} A_{1}\\ B_{1}\\ A_{2}\\ B_{2} \end{array}\right]=D_{b}\left[\begin{array}{l} A_{1}\\ B_{1}\\ A_{2}\\ B_{2} \end{array}\right].$$

In the matrix, *A_1_*, *B*_1_, *A*_2_, and *B*_2_ are the undetermined constants described above. There existed a distinct correlation between the force and displacement experienced for the inner and outer sides of the roller end. The conversion matrix *C* was established, and the expression for *C* is as follows:

(18) $$C=D_{b}D_{a}^{-1}.$$

The boundary conditions are as follows:

The rotating sonotrode was designed as a whole, which meant that the force and displacement were continuous across the entire rotating sonotrode. The following coupling relationship that exists between the thin end and the roller end of the rotating sonotrode is as follows:

(1) The axial displacement of the thin end is equal to the deflection of the roller end, namely

(19) $$u_{1}=w_{r|r=R_{a}}.$$

(2) The axial force in the thin end is equal to the shear force in the roller end, namely

(20) $$F_{1}=Q_{r|r=R_{a}}.$$

(3) The rotation angle of the roller end at the junction is zero, which means that

(21) $$\beta_{r|r=R_{a}}=0.$$

When the rotating sonotrode is in a free state, the following boundary conditions are applied to the entire rotating sonotrode:

(4) The axial input force of the rotating sonotrode is zero, and the bending moment and shear force outside the roller end are also zero, which means that

(22) $$F_{0}=0,$$

(23) $$M_{r|r=R_{b}}=0,$$

(24) $$Q_{r|r=R_{b}}=0.$$

According to the boundary conditions, the force and displacement at the junction between the thin end and the roller end were continuous. Equation (1) can be upgraded to a fourth-order matrix. The fourth-order matrix is as follows:

(25) $$\left[\begin{array}{l} w_{r|r=R_{a}}\\ \beta_{r|r=R_{a}}\\ M_{r|r=R_{a}}\\ Q_{r|r=R_{a}} \end{array}\right]=\left[\begin{array}{cccc} \cos kL_{1} & 0 & 0 & -\frac{\sin kL_{1}}{kES_{1}}\\ 0 & 1 & 0 & 0\\ 0 & 0 & 1 & 0\\ kES_{1}\sin kL_{1} & 0 & 0 & \cos kL_{1} \end{array}\right]\left[\begin{array}{c} u_{0}\\ \beta_{r|r=R_{a}}\\ M_{r|r=R_{a}}\\ F_{0} \end{array}\right].$$

According to Relationship (17), a matrix between the force and displacement for the roller end can be derived. The expression for this is as follows:

(26) $$\left[\begin{array}{l} w_{r|r=R_{b}}\\ \beta_{r|r=R_{b}}\\ M_{r|r=R_{b}}\\ Q_{r|r=R_{b}} \end{array}\right]=C\left[\begin{array}{cccc} \cos kL_{1} & 0 & 0 & -\frac{\sin kL_{1}}{kES_{1}}\\ 0 & 1 & 0 & 0\\ 0 & 0 & 1 & 0\\ kES_{1}\sin kL_{1} & 0 & 0 & \cos kL_{1} \end{array}\right]\left[\begin{array}{c} u_{0}\\ \beta_{r|r=R_{a}}\\ M_{r|r=R_{a}}\\ F_{0} \end{array}\right].$$

The total transfer matrix is *Z*, and the expression of *Z* is as follows:

(27) $$Z=C\left[\begin{array}{cccc} \cos kL_{1} & 0 & 0 & -\frac{\sin kL_{1}}{kES_{1}}\\ 0 & 1 & 0 & 0\\ 0 & 0 & 1 & 0\\ kES_{1}\sin kL_{1} & 0 & 0 & \cos kL_{1} \end{array}\right].$$

According to the boundary conditions outlined in Formulas (20)–(23), the frequency equation of the rotating sonotrode can be obtained. The frequency equation is as follows:

(28) $$\left[\begin{array}{cc} Z_{31} & Z_{33}\\ Z_{41} & Z_{43} \end{array}\right]=0.$$

### 3.2. Relevant Parameters and Calculation Methods

The relevant parameters required in this study are shown in Table 1. TC4 titanium alloy has high acoustic velocity, yield strength, and fatigue strength [38,39], which can better meet the needs of rolling weld edge and high-frequency vibration. The material utilized for the rotating sonotrode was TC4 titanium alloy. The length of each fine end of the rotating sonotrode was half a wavelength. In order to avoid the loss of axial vibration caused by radial vibration during the transmission of ultrasonic vibration, the thin end should have a diameter less than a quarter of the wavelength. In order to ensure the strength of the thin end after applying pressure, its radius was set to 10–15 mm. In order to ensure the slotting requirements and mechanical strength of the roller end, the width range of the roller end was set to 16–20 mm in this study. The roller end of the rotating sonotrode was regarded as a medium-thick plate, and the radius of the roller end was set to 40 mm in accordance with the thickness–diameter ratio of the medium thick plate.

The frequency equation was solved to determine the return value that is close to the base value by taking the parameters in Table 1 as the base value. Two methodologies were employed in the numerical solution. One approach involved the utilization of discrete basis values, wherein the thin-end radius, the radius, and thickness of the roller end were combined into a group of basis values, and the return value of each group of basis values was determined. Another approach involved the utilization of an interval-based solution, wherein the radius range of the thin end was defined as an interval, and the radius range and thickness range of the roller end was defined as an interval. The return value was found within the three intervals. Combined with the results from the two approaches, the size of rotating sonotrode with a low machining accuracy was finally obtained. In order to determine the return value with the smallest frequency error, the characteristic frequency under different return values was analyzed by finite element simulation.

### 3.3. The Size and Characteristic Frequency of the Unslotted Rotating Sonotrode

The frequency Equation (28) is solved using the discrete base-value cable solution method. In taking the solution result as the size of the rotating sonotrode, a characteristic frequency analysis was carried out using the finite element simulation method, and the results are shown in Table 2. When the radius of the thin end was 11.6395 mm, the radius of the roller end was 39.6248 mm; the thickness of the roller end was 17.0428 mm; the characteristic frequency of the rotating sonotrode was 49.026 kHz, which is close to the target design frequency; and the frequency error was 1.948%.

The results obtained based on the interval-based solution and the simulation results of the characteristic frequency are shown in Table 3. When the radius of the thin end was 13.3 mm, the radius of the roller end was 40 mm, the thickness of the roller end was 18.6 mm, and the frequency error was 0.004%, the characteristic frequency of the rotating sonotrode was 49.998 kHz, which is close to the target design frequency. The vibration mode is the longitudinal bending resonance mode.

In comparing the results based on discrete basis values and interval-based solutions, the results that were based on discrete basis values had a higher accuracy, while the dimensional accuracy was closely related to the machining difficulty of the rotating sonotrode. The higher the dimensional accuracy, the more difficult the machining. According to the above calculation results, when the radius of the thin end was 13.3 mm and the radius and thickness of the rolling end were 40 mm and 18.6 mm, respectively, the dimensional accuracy was small, and the error between the characteristic frequency and the target design frequency was minimal. Therefore, the size was finally determined to be the size of the unslotted rotating sonotrode.

## 4. Design and Optimization of the Slotted Rotating Sonotrode

### 4.1. The Influence of Slotting on the Vibration Mode and Characteristic Frequency

**Figure 4:**
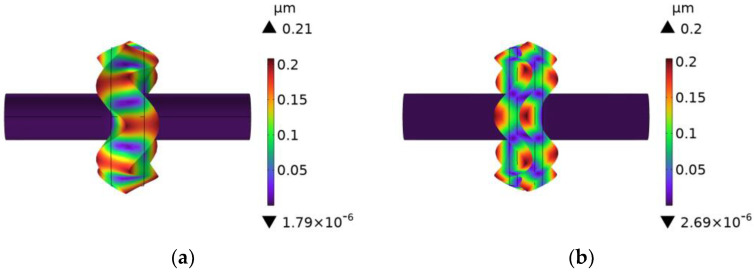
The vibration mode of the rotating sonotrode: (**a**) the unslotted rotating sonotrode and (**b**) the slotted rotating sonotrode.

Based on the calculated unslotted rotating sonotrode size, in order to meet the design goal where the rotating sonotrode can roll on the base material on both sides of the weld during the welding process, a semicircular groove should be established around the roller end, as shown in Figure 1b. The semicircular groove had a radius of 5 mm. The vibration mode and characteristic frequency of the slotted rotating sonotrode were studied using finite element simulation. The vibration mode diagram of the rotating sonotrode is shown in Figure 4. When the slotted rotating sonotrode maintained the longitudinal–flexural resonance mode, the characteristic frequency of the rotating sonotrode was significantly reduced, and its characteristic frequency was reduced to 45.093 kHz. The error between the characteristic frequency and the target design frequency was 9.816%. In order to enable the slotted rotating sonotrode to attain longitudinal–flexural resonance at the target frequency (50 kHz), it was imperative to implement certain optimization measures to increase the characteristic frequency of the slotted rotating sonotrode while preserving its vibration mode as longitudinal–flexural resonance. When the mode of the sonotrode was the longitudinal bending resonant mode, the amplitude on the output face of the roller end included radial amplitude and axial amplitude, and the radial amplitude was larger than the axial amplitude.

### 4.2. The Influence of the Transitional Form on the Stress Distribution

The service life of the rotating sonotrode was easily affected by the stress concentration at the junction of the roller end and the thin end. When the rotating sonotrode works for a long time, it is easy to have fatigue fracture at the junction. In order to decrease the stress concentration at the junction of the roller end and the thin end, four transitional forms were designed at the junction, namely single-curvature arc transition, double-curvature arc transition, three-curvature arc transition, and ellipse transition. In this part, the effect of the different transitional form on the stress distribution at the junction was investigated. According to the existing research results [16], there is an optimal single-curvature arc transition form for the stepped sonotrode. The stress concentration at the variable section can be alleviated while keeping the characteristic frequency basically unchanged. The radius of the single-curvature arc is proportional to the radius of the thin end of the rotating sonotrode. The ratio is related to the ratio of the radius of the roller end to the radius of the thin end. The ratio of the radius between the roller end and the thin end of the rotating sonotrode designed in this study was about 3, and the ratio of the radius of the single-curvature arc to the radius of the thin end is 0.4. For the single-curvature arc, the optimal radius is as follows:

(29) $$R=0.4R_{a}.$$

**Figure 5:**
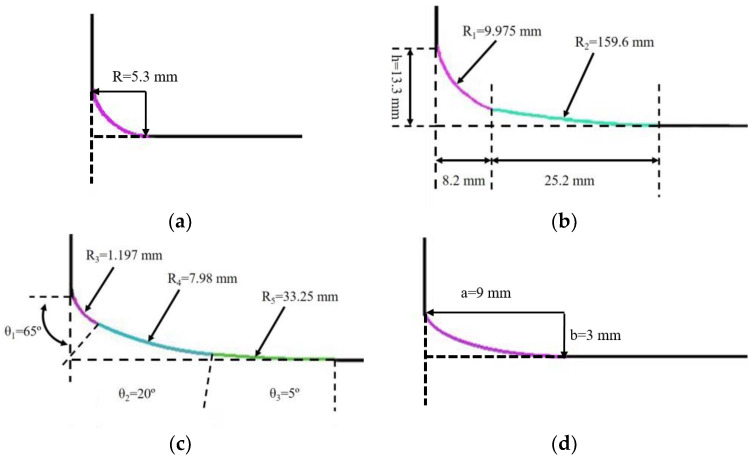
Different transition modes: (**a**) single-curvature arc transition, (**b**) double-curvature arc transition, (**c**) three-curvature arc transition, and (**d**) elliptical transition.

In the formula, *R* and *R_a_* are the radius of the single-curvature arc and the thin end, respectively. According to this formula, the radius of the single-curvature arc was calculated to be 5.3 mm, and the two-dimensional diagram about the single-curvature arc transition is shown in Figure 5a.

The curve expression of the double-curvature arc transition is as follows [18] (where *R*_1_ and *R*_2_ are the radius of the two arcs, respectively, and *h* is the height of the double-curvature arc transition):

(30) $$h=R_{a},$$

(31) $$R_{1}=0.75h,$$

(32) $$R_{2}=16h.$$

By introducing the thin-end radius of the rotating sonotrode into Formulas (30)–(32), the relevant parameters of the double-curvature arc can be obtained. The two-dimensional diagram of the double-curvature arc transition mode is shown in Figure 5b.

The three-curvature arc transition curve is expressed as follows [18] (where *R*_1_, *R*_2_, and *R_3_* represent the radius of the three arcs, respectively, and *θ*_1_, *θ*_2_, and *θ*_3_ represent the angles of the three arcs, respectively):

(33) $$R_{3}=0.09R_{a},\;\;\;\;\;\theta_{1}=65^{{}^{\circ}},$$

(34) $$R_{4}=0.6R_{a},\;\;\;\;\;\theta_{2}=20^{{}^{\circ}},$$

(35) $$R_{5}=2.5R_{a},\;\;\;\;\;\theta_{3}=5^{{}^{\circ}}$$

By substituting the radius of the thin end of the rotating sonotrode into Formulas (33)–(35), the relevant parameters of the three-curvature arc can be obtained. The two-dimensional diagram of the three-curvature arc transition mode is shown in Figure 5c.

According to reference [18], the elliptical transition mode exhibits the most effective effect on reducing stress concentration when the ratio between the long and short axes of the ellipse (a/b) is equal to the ratio between the roller end radius and the thin-end radius of the rotating sonotrode. Hence, the length axis of the ellipse was set to 9 mm, and the short axis was set to 3 mm. The two-dimensional diagram of the elliptical transition section is shown in Figure 5d.

The simulation of the slotted rotating sonotrode with four different transition modes was carried out using finite element simulation. The simulation results showed that the characteristic frequency of a rolling sonotrode with the double-curvature arc transition mode is greatly changed, and that its characteristic frequency with other transition modes changes little. The characteristic frequency of a sonotrode is its inherent property. In the case of the same material being used, the characteristic frequency is mainly affected by the shape. The height and length were large for the double-curvature arc transition section, which caused the shape of the sonotrode to change greatly. Therefore, the characteristic frequency of the sonotrode varied greatly compared with the other transition modes. The results of the characteristic frequency are presented in Table 4, and the vibration mode used was the longitudinal–flexural resonance one.

**Figure 6:**
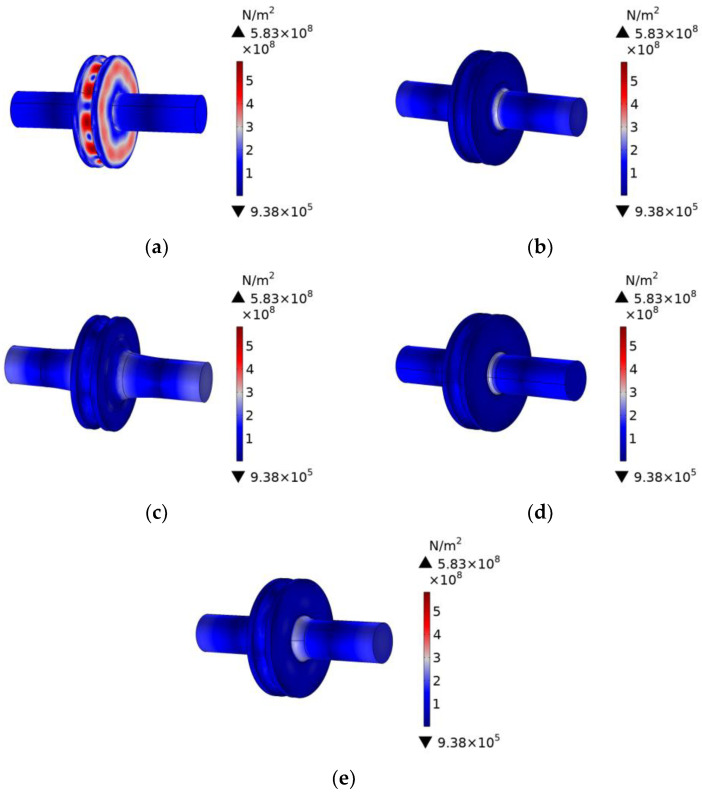
Diagram of stress distribution under different transition modes: (**a**) no transition, (**b**) single-curvature arc transition, (**c**) double-curvature arc transition, (**d**) three-curvature arc transition, and (**e**) elliptical transition.

The two thin ends of the rotating sonotrode were impacted with ultrasonic amplitudes of 10 µm, and the stress distributions of the rotating sonotrode with different transition modes were analyzed at their respective characteristic frequencies. The results are presented in Figure 6. When there was no transition section, the stress on the roller end was larger than that on the thin end. The stress concentration occurred at the junction between the roller end and the thin end. During a long time of vibration, the rotating sonotrode was easy to break at the stress concentration position. After the transition section was established, the stress distribution of the rotating sonotrode was changed and the stress at the roller end had evidently decreased. The maximum stress was mainly located at the transition position between the roller end and the thin end. The interface between the transition section and the roller end was defined as the inner interface, and the Interface between the transition section and the thin end was defined as the outer interface. The peak, minimum, and mean values of stress on the two interfaces under different transition modes are shown in Table 5.

For the single-curvature arc, three-curvature arc, and elliptic transitions, the stress at the junction between the transition section and the thin end was greater than that at the junction between the transition section and the roller end. However, for the stress at the junction between the transition section and the thin end, the stress under the three-curvature arc and ellipse transition mode was less than that under the single-curvature arc transition mode. This was because the transition section of the three-curvature arc transition and the elliptic transition was longer, and the curvature was also much smaller than that of the single-curvature arc. The stress transition was more gentle, which further alleviated the stress at the junction of the transition segment and the thin end. For the double-curvature arc transition, the stress at the junction between the transition section and the thin end was small, while the stress at the junction between the transition section and the roller end was large. This was because the radius and length of the first circular arc were much smaller than that of the second circular arc, and the curvature of the first circular arc was greater than that of the second circular arc for the double-curvature arc transition curve, such that the stress at the junction between the transition section and the roller end was greater than that at the junction between the transition section and the thin end.

**Figure 7:**
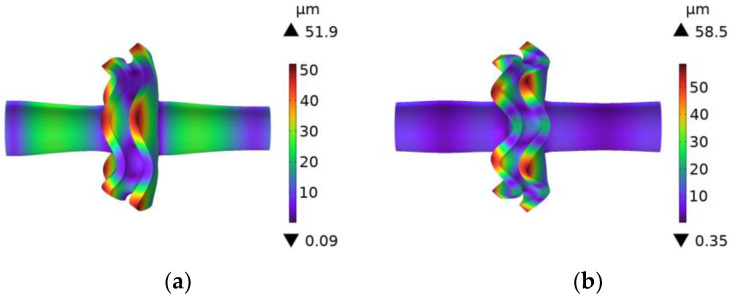
A vibration mode and amplitude distribution cloud diagram of the rotating sonotrode with elliptical transition: (**a**) fifth order and (**b**) sixth order.

Compared to the single-curvature arc, the three-curvature arc transition and the elliptical transition had a significant effect on the decrease in stress concentration at the junction of the rotating sonotrode. However, the design of the three-curvature arc transition section was more complicated and the size of the transition section was small, which was not convenient for the machining. Therefore, this study chose the elliptical transition as the transition mode of the rotating sonotrode. The amplitude distribution and vibration mode of the rotating sonotrode with the elliptical transition was analyzed, and the result is shown in Figure 7. The results showed that the ultrasonic amplitude at the outer edge of the roller end had a maximum value of 51.9 µm.

### 4.3. The Influence of the Size and Position of the Perforation on the Characteristic Frequency and Amplification Coefficient

**Figure 8:**
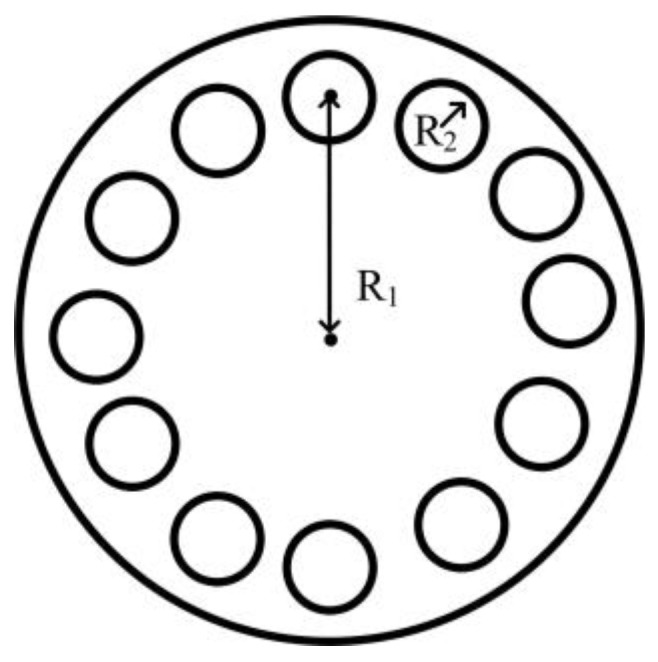
Diagram of the perforation on the roller end.

In order to ensure that the slotted rotating sonotrode had a longitudinal–flexural resonance mode and a target design frequency, some perforations were designed for the rolling sonotrode. It has been shown that perforation will reduce the characteristic frequency of a sonotrode [40,41], and the magnification factor of a sonotrode can be improved by designing a suitable perforation scheme. In this paper, the effect of perforation position and size on characteristic frequency and amplification was analyzed. The number of perforation was fixed to 12, and the radius and position of the perforation were changed. The, respective, radius of the perforations were 1 mm, 2 mm, and 3 mm. The positions of the perforations were determined by the distances between the center of the perforation to the axis of the roller end. The positions were, respectively, 24 mm, 28 mm, 32 mm, and 36 mm. The diagram is shown in Figure 8.

**Figure 9:**
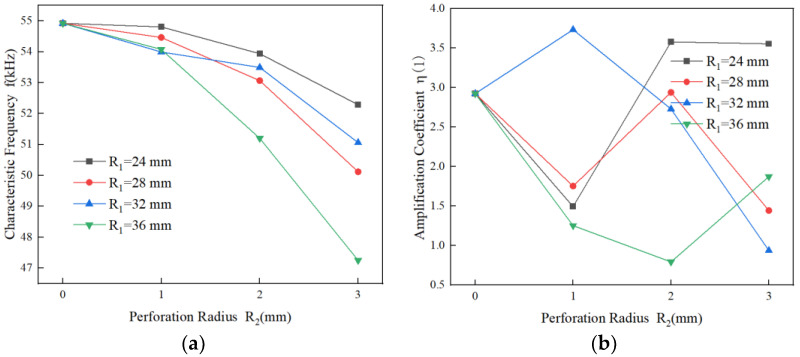
The relationship between the perforation and the characteristic frequency and amplification coefficient of the rotating sonotrode (sixth order): (**a**) characteristic frequency and (**b**) amplification coefficient.

The analysis of the characteristic frequency and amplification coefficient of the rotating sonotrode with various perforation options was conducted using finite element simulation. The amplification coefficient is the ratio of the ultrasonic amplitude of the roller end to that of the thin end. A 10 µm ultrasonic amplitude was applied to the end face of the fine end. The frequency of the rotating sonotrode with the longitudinal–flexural resonance mode was analyzed. The relationship between the characteristic frequency, amplification coefficient, and the size and position of the perforation is shown in Figure 9.

The results were similar to those of previous studies, whereby the characteristic frequency of the rotating sonotrode was reduced and the amplification factor was also changed. The characteristic frequency of the rotating sonotrode generally decreased with increases in the size of the perforation. Compared with the characteristic frequency of the rotating sonotrode without perforation, the characteristic frequency of the rotating sonotrode with perforation increased. The amplification coefficient changed irregularly. The characteristic frequency decreased and the amplification coefficient changed irregularly when the perforation position was away from the axis.

### 4.4. The Influence of the Double-Position Joint Perforation on the Characteristic Frequency and Amplification Coefficient

**Figure 10:**
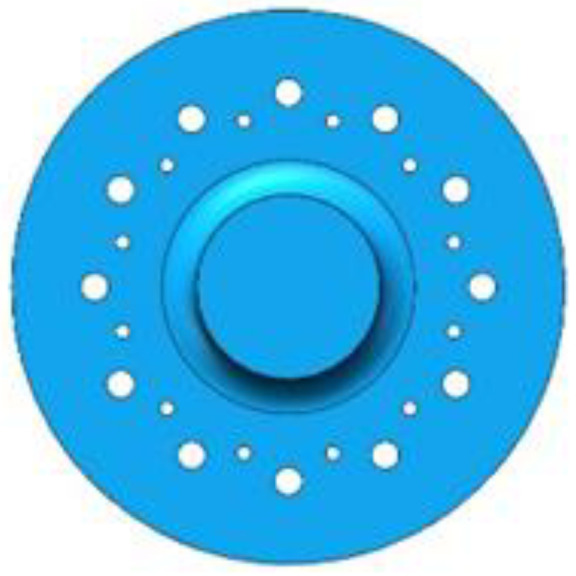
Schematic diagram of the two-position joint perforation.

As can be seen from Figure 9, when the position of the perforation was 24 mm and the radius was 2 mm or 3 mm, there was a large amplification coefficient. When the position of the perforation was 28 mm and the radius was 2 mm or 3 mm, the characteristic frequency was closer to the target design frequency, and the amplification factor was moderate. In this study, in order to make the rotating sonotrode have the target design frequency and large amplification coefficient, a double-position joint perforation scheme was proposed, as shown in Figure 10. The position of the perforation was divided into inner perforation and outer perforation. The position of the inner perforation was 24 mm from the axis of the roller end, the position of the outer perforation was 28 mm from the center of the roller end, and the radius of the perforation was 2 mm or 3 mm. The characteristic frequencies and amplitudes of the rotating sonotrode with different perforation schemes were analyzed by the finite element method. The simulation results are shown in Table 6.

The characteristic frequency of the rotating sonotrode was evidently reduced and the amplification coefficient of the rotating sonotrode was clearly increased by using the double-position joint perforation scheme. The double-position joint perforation includes inner perforation and outer perforation. The number of perforations is increased to further reduce the characteristic frequency, and the inner perforation (R_1_ = 24 mm) is used to increase the amplification factor of the rotating sonotrode.

**Figure 11:**
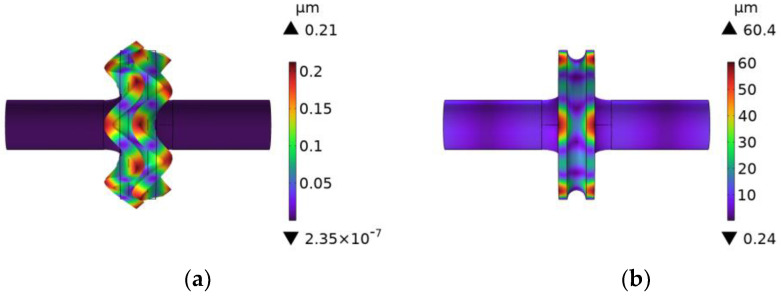

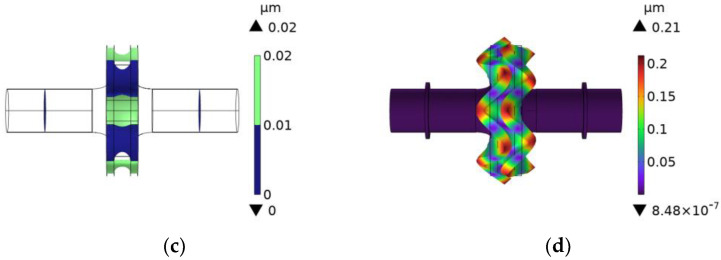
The characteristic frequency, amplitude cloud diagram, and wave section of the rotating sonotrode with an inner perforation radius (3 mm), an outer perforation radius (2 mm), and a distance of 28 mm in the outer perforation’s place. (**a**) Vibration mode, (**b**) amplitude cloud diagram, (**c**) wave node, and (**d**) a vibration mode diagram with flanges.

According to Table 6, when the inner perforation’s radius was 3 mm and the outer perforation’s radius was 2 mm, the characteristic frequency was in close proximity to the target design frequency, and the frequency error was only 0.558%. Within the allowable range of error (5%), it had a large amplification coefficient of 3.02. The vibration mode, amplitude cloud diagram, and wave node position are depicted in Figure 11.

The position of the wave nodes was clearly visible on the thin end of the rotating sonotrode with the multi-position joint perforation. The wave node’s position was near the middle of the thin end. In the position of the wave node, a flange with a width of 2 mm was implemented. After adding the flange, the characteristic frequency of the rotating sonotrode was 49.733 kHz. In contrast to the rotating sonotrode without a flange, the frequency variation of the rotating sonotrode was very small, the vibration mode of which is depicted in Figure 11d.

The rotating sonotrode designed in this study has the following advantages: (1) A design method for a rotating sonotrode with a groove end and double-thin-end structure. The error between the frequency of the designed sonotrode and the target frequency is small, and the dimensional machining accuracy is low. (2) The length of the sonotrode is 140.6 mm, the width of the roller end is 18.6 mm, and the acoustic sonotrode has the advantage being miniaturized. (3) The acoustic sonotrode can roll on both sides of the weld by grooving on the roller end. The node of the acoustic sonotrode is located in the middle of the thin end, which facilitates the installation of the flange to complete the fixing of the acoustic sonotrode.

The main limitation of this study is that the groove size of the roller end is only suitable for a specific-sized weld. The groove size designed in this study was proposed based on the weld size of previous studies [7], which is only applicable to single-layer welds with a weld width of less than 10 mm, and it cannot be used for welding when operating with a large weld width. If the groove size needs to be changed, it must be designed according to the design method proposed in this paper.

## 5. Conclusions

In this paper, a rotating sonotrode for the ultrasonic vibration-assisted arc welding of metal materials was designed by combining theoretical calculation with finite element simulation. An effective design method is proposed for a rotating sonotrode with a groove and double-thin-end structure. The effects of the groove, perforation, and transition types on the characteristic frequency, stress distribution, and amplification factor of the rotating sonotrode were clarified. The main conclusions are as follows:

(1) For the calculation of the size of the rotating sonotrode without grooving, a numerical solution based on discrete intervals can obtain design results with a lower frequency error and dimensional accuracy than a numerical solution based on discrete basis values. The fifth-order mode and its characteristic frequency are obtained by finite element simulation. The longitudinal bending resonant mode is used, and the frequency error is only 0.004%. After the rolling end is grooved, the characteristic frequency of the rotating sonotrode clearly decreases.

(2) The transition mode of the connection between the roller and the thin end has a significant impact on the stress distribution and characteristic frequency of the sonotrode. The elliptic transition mode can significantly reduce the stress concentration of the sonotrode without changing the characteristic frequency of the sonotrode.

(3) The perforation on the roller end will affect the characteristic frequency and amplification factor of the rotating sonotrode. The characteristic frequency of the sonotrode decreases with increases in the size of the perforation. When the perforation size is constant, the characteristic frequency of the sonotrode also decreases with the perforation position away from the axis of roller end. The amplification factor changes irregularly. Through the coupling effect of the perforation size and position on the characteristic frequency and amplification factor of the sonotrode, a rotating sonotrode with a characteristic frequency of 49.721 kHz and an amplification factor of 3.02 is obtained by the method of a joint perforation in two positions, which meets the design requirements.

## Figures and Tables

**Table 1:** The related parameters.

Parameter Name/Symbol	Numerical Value
The length of the thin ends on both sides of the rotating sonotrode/*L*_1_ and *L*_2_	61 mm
Rotating sonotrode radius/*R_a_*	10–15 mm
Roller end radius of rotating sonotrode/*R_b_*	40 mm
Thickness of the roller end of rotating sonotrode/*T*	16–20 mm
Density of the TC4 titanium alloy/*ρ*	4.43 g/cm^3^
Elastic modulus of the TC4 titanium alloy/*E*	110 G Pa
Poisson’s ratio of the TC4 titanium alloy/*μ*	0.34

**Table 2:** The cable solution results and simulated values of the characteristic frequencies based on discrete basis values.

	Number of Classes				
	1	2	3	4	5
Thin-end radius (mm)	10.8735	11.6395	12.9620	13.5615	14.1484
Radius of the roller end (mm)	37.9179	39.6248	37.8928	41.5578	43.1557
Thickness of the roller end (mm)	20.2326	17.0428	20.3324	19.9792	16
Entry 4 characteristic frequency (kHz)	55.23	49.026	55.368	48.667	54.073
Frequency error (%)	10.46	1.948	10.736	2.666	8.146

**Table 3:** Cable solution results based on the interval-based solution.

	Number of Classes						
	1	2	3	4	5	6	7
Thin-end radius (mm)	11.6	13.3	13.3	13.3	13.5	13.6	13.8
Radius of the roller end (mm)	39.6	40	40	40	39.5	41.6	39.4
Thickness of the roller end (mm)	17.1	18.6	18.7	18.8	18.5	20	18.5
Characteristic frequency (kHz)	48.621	49.998	50.101	50.187	50.778	48.621	50.949
Frequency error (%)	2.758	0.004	0.202	0.374	1.556	2.758	1.898

**Table 4:** Characteristic frequencies of the rotating sonotrode with different transition modes.

Transition Mode	Characteristic Frequency (kHz)
No transition	45.093
Single-curvature arc transition	45.282
Double-curvature arc transition	48.172
Three-curvature arc transition	45.217
Elliptical transition	45.226

**Table 5:** The stress magnitude of the two interfaces with different transition modes.

	Interface	No Transition	Single-Curvature Arc	Double-Curvature Arc	Three-Curvature Arc	Ellipse
Peak stress (N/m^2^)	Inner side	2.74 × 10^8^	1.14 × 10^8^	1.89 × 10^8^	4.22 × 10^7^	6.36 × 10^7^
	Outer side		2.4 × 10^8^	3.54 × 10^7^	1.46 × 10^8^	1.71 × 10^8^
Minimum stress (N/m^2^)	Inner side	1.93 × 10^8^	8.55 × 10^7^	1.26 × 10^8^	8.03 × 10^6^	3.76 × 10^7^
	Outer side		2.2 × 10^8^	2.86 × 10^7^	1.43 × 10^8^	1.65 × 10^8^
Mean stress (N/m^2^)	Inner side	2.23 × 10^8^	9.98 × 10^7^	1.55 × 10^8^	2.58 × 10^7^	5.04 × 10^7^
	Outer side		2.28 × 10^8^	3.2 × 10^7^	1.45 × 10^8^	1.67 × 10^8^

**Table 6:** Characteristic frequency and amplification coefficient of the rotating sonotrode with different perforation distributions.

Inner Perforation	Outer Perforation	Characteristic Frequency (kHz)	Amplification Coefficient
The perforation radius was 2 mm.	The perforation radius was 2 mm.	51.988	3.18
	The perforation radius was 3 mm.	49.598	3.185
The perforation radius was 3 mm.	The perforation radius was 2 mm.	49.721	3.02

## Data Availability

Data are contained within the article.
